# Impact of Node Speed on Energy-Constrained Opportunistic Internet-of-Things with Wireless Power Transfer

**DOI:** 10.3390/s18072398

**Published:** 2018-07-23

**Authors:** Seung-Woo Ko, Seong-Lyun Kim

**Affiliations:** 1 Department of Electrical and Electronic Engineering, The University of Hong Kong, Pok Fu Lam, Hong Kong, China; swko@eee.hku.hk; 2 School of Electrical and Electronic Engineering, Yonsei University, 50 Yonsei-Ro, Seodaemun-Gu, Seoul 03722, Korea

**Keywords:** internet-of-things, opportunistic networks, wireless power transfer, inter-meeting time, Markov chain, node speed, battery capacity, node density

## Abstract

*Wireless power transfer* (WPT) is a promising technology to realize the vision of *Internet-of-Things* (IoT) by powering energy-hungry IoT nodes by electromagnetic waves, overcoming the difficulty in battery recharging for massive numbers of nodes. Specifically, *wireless charging stations* (WCS) are deployed to transfer energy wirelessly to IoT nodes in the charging coverage. However, the coverage is restricted due to the limited hardware capability and safety issue, making mobile nodes have different battery charging patterns depending on their moving speeds. For example, slow moving nodes outside the coverage resort to waiting for energy charging from WCSs for a long time while those inside the coverage consistently recharge their batteries. On the other hand, fast moving nodes are able to receive energy within a relatively short waiting time. This paper investigates the above impact of node speed on energy provision and the resultant throughput of energy-constrained opportunistic IoT networks when data exchange between nodes are constrained by their intermittent connections as well as the levels of remaining energy. To this end, we design a two-dimensional Markov chain of which the state dimensions represent remaining energy and distance to the nearest WCS normalized by node speed, respectively. Solving this enables providing the following three insights. First, faster node speed makes the inter-meeting time between a node and a WCS shorter, leading to more frequent energy supply and higher throughput. Second, the above effect of node speed becomes marginal as the battery capacity increases. Finally, as nodes are more densely deployed, the throughput becomes scaling with the density ratio between mobiles and WCSs but independent of node speed, meaning that the throughput improvement from node speed disappears in dense networks. The results provide useful guidelines for IoT network provisioning and planning to achieve the maximum throughput performance given mobile environments.

## 1. Introduction

Wireless mobile devices are currently pervasive, and the number of the devices is expected to be ever-growing when *Internet-of-Things* (IoT) and smart cities emerge in the near future [1]. This tendency makes their energy supply required not only huge but also so frequent that the existing wired charging technologies cannot cope with them. Faced with the energy supply problem, *wireless power transfer* (WPT) is fast becoming recognized as a viable solution [2] enabling the recharging of batteries without plugs and wires if there is an apparatus to perform WPT, known as a *wireless charging station* (WCS). However, due to the limited capability of the state-of-art WPT technique and concerns about human safety [3], it is impractical to radiate electromagnetic waves with higher power from a WCS, making the charging coverage restricted. Multiple numbers of WCSs can be installed to cover the entire network area, but excessive deployment cost is required.

**Figure 1:**
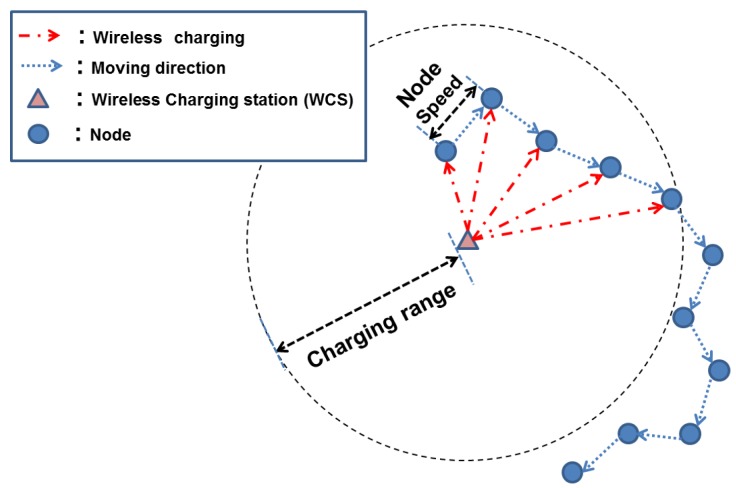
The pattern of wireless charging when node speed is slow. During the period that a node is in the charging coverage of the WCS, it receives energy from WCS continuously. Once a node is out of the charging range, on the other hand, it takes a long time to receive energy from WCS again.

This paper addresses the energy provisioning issue of *energy-constrained opportunistic IoT networks* (This is originated from an opportunistic IoT network where IoT nodes exchange information via *device-to-device* (D2D) communications based on their interaction [4,5]). These have been extensively studied in a wide range of fields, e.g., healthcare, logistics, and car navigation. We add the term “energy-efficient” to highlight the energy provision problem where nodes’ opportunistic connections to other nodes and WCSs lead data transmission and energy charging, respectively. These features of energy supply and consumption yield the following energy dynamics, which is the main theme of this work. Particularly, we pay attention to the random mobility of an IoT node followed by its moving pattern, making it possible to supply energy in spite of WCSs’ limited charging coverage. In other words, a WCS can transfer energy to nodes who get into the charging coverage, which is referred to as “meeting” throughout the paper. The resultant energy provision of the IoT node thus depends on its meeting pattern, dominantly affected by moving speed as shown in Figure 1. When a node moves slowly, as an example, it remains in the charging coverage of the WCS and can receive energy continuously. Once out of the coverage, on the other hand, it may take an extremely long time to receive energy again. Consequently, such an irregular energy provision occurs where some devices consistently receive energy while others suffer from the lack of energy. As a node moves faster, this energy-starving duration is likely to be shorter, leading to a more regular pattern of energy supply. This difference motivates us to investigate the relation between speed, energy provision and the resultant throughput.

### 1.1. Wireless Power Transfer

WPT is a key enabler to realize the vision of next-generation mobile networks, e.g., IoT and smart cities by overcoming the challenge with battery charging. With the aid of WPT, it is possible to deploy thousands of IoT sensors at a low cost. In addition, WPT along with energy harvesting enables the facilitating of designing green networks (see, e.g., [6,7]). Due to its promising potential and the interdisciplinary nature, many new research issues arise in the area of WPT and are widely studied in different fields. Recent advancements in the area can be found in numerous surveys such as [8].

The most widely-used WPT method is the magnetic inductive coupling that electric power is delivered by means of an induced magnetic field. The drawback of this method is its power transfer efficiency that diminishes significantly unless the transmitter and the receiver are close in contact. Recently, there have been efforts to develop WPT technology of which the efficiency remains high within the range from several to tens of meters. In [9], Kurs et al. suggest a novel method called magnetic resonant coupling where electric power is transferred from one to the other with high efficiency when two devices are tuned to the same resonant frequency. However, its high efficiency requirement is so tight that it is vulnerable to the misalignment between a transmitter and a receiver especially when the distance between the two becomes larger. Some sophisticated tracking and alignment techniques are proposed for practical use, e.g., frequency matching [10], impedance matching [11] and resonant isolation [12], but the use of magnetic resonant coupling for long-range battery charging in harsh mobile environments like vehicular scenarios is still doubtful. Another approach is microwave power transfer where *radio-frequency* (RF) waves are delivered to recharge the battery by using advanced techniques of wireless communications, e.g., directional beamforming [13], backscatter communication [14,15], and full duplex communication [16]. Recent studies on WPT consider practical factors to design and optimize realistic systems, e.g., imperfect channel state information [17], nonlinear energy harvesting efficiency [18,19], and waveform design [20]. Microwave power transfer theoretically enlarges the charging coverage more than tens of meters, but controversial issues on health impairments caused by RF exposure have not been resolved yet, making it difficult to use commercially.

### 1.2. Applying Wireless Power Transfer to Wireless Networks

There have been several studies incorporating WPT into the design of energy-constrained wireless networks. One research thrust focuses on the design of the efficient recharging protocol to make every node always active. For example, a *wireless charging vehicle* (WCV) is suggested in [21] that visits all nodes to recharge their batteries. The optimal travel path is derived to avoid the battery depletion of each node. The work of the optimal WCV routing is generalized in [22] such that the battery charging is enabled in every place in the entire network under the consideration of the trade-off between charging efficiency and distance. In [23], the optimal routing for safe charging problem is studied where no location in the networks has electromagnetic radiation exceeding a given threshold. A prototype testbed of the routing platform is constructed by using off-the-shelf RF energy transfer hardware equipment in [24] to demonstrate the performance of wireless sensor networks powered by RF energy transfer. A distributed recharging protocol is proposed in [25] where multiple WCVs wirelessly provide energy to nodes given the limited network information. The concept of Qi-ferry is introduced in [26], which is similar to WCV except the fact that Qi-ferry consumes its own residual energy when it is moving. In other words, longer travel distance of Qi-ferry visits more nodes but accelerates its energy depletion. They optimize its travel path reflecting the above trade-off. Nevertheless, these papers [21,22,23,24,25,26] are based on the assumption that WPT-enabled devices have knowledge of full or limited geographical information for all rechargeable nodes, hardly estimated in mobile environments.

Recently, there have been some trials to exploit node mobility for battery charging in WPT-aided mobile networks. In [27], the energy provision based on the node mobility is introduced where nodes can harvest excessive energy in a power-rich area and store it for later use in a power-deficient area. The number of necessary WCSs for continuous operation of every node is analyzed, but some practical aspects like node speed and battery capacity are ignored. In [28], the performance of energy-constrained mobile networks is analyzed using stochastic geometry assuming that the energy arrival process of each node as an *independent and identically distributed* (i.i.d.) sequence, which is reasonable only when the speed of each node is extremely fast. Delay-limited and delay-tolerant communications with WPT are respectively studied in [29,30], where a node can move to a few rechargeable points according to predetermined transition probabilities. In [31], an intentional mobility to WPT-enabling locations for battery charging, called a spatial attraction, is modeled as a Markov chain and analyzed to show the improvement of the coverage rate by the optimally controlled power and charging range. These papers [27,28,29,30,31] do not consider node speed in spite of its significant effects on the energy arrival process. In [32], a *quality of energy provisioning* (QoEP) is defined as the expected portion of time a node sustains its operation when mobiles are moving within a given range of node speed. It is shown that QoEP converges to one as battery capacity or node speed increases. The analytical results are based on the continuous transmission model where a node keeps transmitting data whenever it has energy. In IoT networks, on the other hand, data is transmitted discontinuously according to a few specific conditions, making it more challenging to analyze.

### 1.3. Contributions and Organization

In this work, we study the performance of energy-constrained opportunistic IoT networks where the opportunistic behaviors of mobile nodes affect the patterns of data transmission and energy charging. Specifically, data delivery is enabled between nodes when (1) they are intermittently connected and (2) a transmitting node receives energy from WCSs enough to deliver data. To reflect the above energy dynamics, we design a two-dimensional Markov chain of which the horizontal and vertical state dimensions represent the remaining energy and the distance to the nearest WCS, respectively. We derive its steady-state probabilities and aim at explaining the effect on throughput. The main contributions of this paper are summarized below.

- **Inter-meeting time vs. Throughput**: Higher node speed reduces the frequency of lengthy inter-meeting times between a node and a WCS and eventually improves the throughput. The inter-meeting time is interpreted as an energy-starving duration. We explain the phenomenon through the stochastic distribution of the inter-meeting time in Proposition 1.
- **Node speed vs. battery capacity**: A slow-moving node stays in the charging coverage for a long time. It saves enough energy to endure a lengthy inter-meeting time if its battery capacity, the maximum amount of energy stored in the battery, is large enough. In Proposition 2, we show that a fast-moving node achieves the same throughput when the battery capacity becomes infinite.
- **Throughput scaling law**: In Proposition 3, we prove that the throughput scaling is given as $\Theta \left(\min\left(1,\frac{m}{n}\right)c^{\min\left(1,\frac{m}{n}\right)}\right)$ (We recall that the following notation: (i) f(n)=O(g(n)) means that there exists a constant *c* and integer *N* such that f(n)≤cg(n) for n>N. (ii) f(n)=Θ(f(n)) means that f(n)=O(g(n)) and g(n)=O(f(n)).) where *n* and *m* denote the number of nodes and WCSs respectively, and *c* is a constant (0<c<1). As the network becomes denser, the throughput depends on the ratio $\frac{m}{n}$ and becomes independent of node speed.

Note that the approach in this work is similar to that of our previous work [33] as both apply a Markov chain to model an energy-constrained mobile network. In [33], it is assumed that nodes follow the i.i.d. mobility model, which allows us to include only the residual energy status as a Markov chain state. On the other hand, our current work focuses on finite node speed, which limits node movement within a restricted area. In other words, the current node location depends on the previous one. Therefore, we should take into account not only the residual energy, but also the location information of a node when designing a Markov chain model. Our paper illustrates that the throughput under the i.i.d. mobility model in [33] can be understood as an upper bound, which is achievable when (i) node speed becomes faster; (ii) battery capacity becomes larger or (iii) node density increases.

The rest of this paper is organized as follows: In Section 2, we explain our models and metrics. In Section 3, we introduce a two-dimensional Markov chain design and derive its steady state probabilities. In Section 4, we verify how the node speed effect is affected by battery capacity and node density. Finally, we conclude this paper in Section 5.

## 2. Models and Metrics

### 2.1. Network Description

Consider an energy-constrained IoT network where *n* nodes and *m* WCSs are randomly distributed in a torus area (A torus area refers to finite and boundary-less region such that one side’s edge is connected to the opposite one. In this model, the boundary effect disappears, enabling the analysis of the performance tractably from the viewpoint of one typical node. ) of size $\sqrt{S}\times \sqrt{S}$ (in meter${}^{2}$). Time is slotted and one slot is large enough to transmit a single packet. A node is assumed to change its direction randomly at every slot with constant speed of *v* (meter/slot), namely, we have:

(1) $$\begin{array}{c} ∥X_{\ell }\left(t+1\right)-X_{\ell }\left(t\right)∥=v, \end{array}$$

where $X_{\ell }\left(t\right)$ is the location of node *ℓ* at slot *t* and ∥·∥ means the Euclidian distance. The assumption makes sense because an IoT node requires much longer latency than conventional cellular networks to transmit data, say up to a few seconds [34].

A node enables transmitting its packet to one of its neighbors within *r* (in meters) defined as the transmission range. For an interference model, we use the well-known protocol model [35] where the packet transmission is successful only when the other transmitting nodes are no less than *r*. Too large *r* leads to frequent transmission failures because there are many interfering nodes. To avoid excessive interference, the transmission range *r* is set to the average distance to the nearest node in the area.

### 2.2. Two-Phase Routing

A pair of source and destination nodes is given randomly. Unless there is the corresponding destination node of a source node within the range *r*, its packet should be delivered via a relay node. This paper adopts the *two-phase routing* [35] as follows:

- *Mode switch.* In the beginning of each slot, a node becomes a transmitter or a receiver with probability *q* or 1−q, respectively. Without loss of generality, we set q=0.5.
- *Phase 1.* In odd slots, let us consider node *ℓ* becomes a transmitter. If there is at least one receiver within transmission range *r*, node *ℓ* forwards its packet to one of them. This receiver node can be the destination of node *ℓ*.
- *Phase 2.* In even slots, let us consider node *ℓ* becoming a receiver. If there is at least one transmitter within transmission range *r* and one of them has a packet whose destination is node *ℓ*, it forwards the packet to node *ℓ*. This transmitter can be the source of node *ℓ*.

In [35], the throughput of the two-phase routing is defined as follows:

**Definition** **1.** *(Throughput) Let $M_{\ell }\left(t\right)$ be the number of node ℓ’s packets that its corresponding destination node receives during t slots. We say that the throughput of *Λ* is feasible for every S-D pair if:*

(2) $$\begin{array}{c} \underset{t\to \infty }{lim inf}\frac{1}{t}M_{\ell }\left(t\right)\geq \Lambda . \end{array}$$

When a node transmits a packet, a constant amount of energy is consumed defined as one *unit of energy* (It is implicitly assumed that a *modulation and coding scheme* (MCS) is fixed and constant power is required to deliver a packet within the transmission range. It is interesting to adjust the level of MCS to improve the energy efficiency, which is outside the scope of current work.). A node is called *active* when it has at least one unit of energy. Otherwise, the node is *inactive*. Let $p_{\mathrm{on}}$ denote the probability that a node is active, defined as an *active probability*. In [33], the throughput Λ is given as follows:

(3) $$\begin{array}{c} \Lambda =\frac{1}{2}qp_{\mathrm{on}}\exp\left(-\frac{\pi }{4}qp_{\mathrm{on}}\right)\left(1-\exp\left(\frac{\pi }{4}\left(-1+q\right)\right)\right). \end{array}$$

It is shown that the throughput Λ (3) depends on $p_{\mathrm{on}}$, which is determined by the process of energy recharging introduced in the sequel.

### 2.3. Recharging Mechanism by Wireless Charging Stations

Inactive nodes are unable to transmit packets in their buffers. To supply energy to them, *m* WCSs are deployed in the network. WCSs recharge nodes via WPT. No interference between data transmission and energy transfer exists because each of them use a separated band.

The energy transferred to a mobile is given by the product of the maximum deliverable units of energy *E* and the energy transfer efficiency τ(x), where *x* is the distance to a WCS. Let $R_{y}$ denote the maximum distance that a node can receive *y* units of energy. Without loss of generality, the efficiency τ(x) is a monotone decreasing function of *x*, and the charging range $R_{y}$ is determined by finding the value of *x* that E·τ(x) becomes *y*, such that $R_{y}=\left\{x:E\cdot \tau \left(x\right)=y\right\}$. Let $Y_{s}\left(t\right)$ denote the location of WCS *s* at slot *t*. The distance between node *ℓ* and WCS *s* is given as $∥X_{\ell }\left(t\right)-Y_{s}\left(t\right)∥$, and the amount of recharged energy υ (in units of energy) is

(4) $$\begin{array}{c} \upsilon (∥X_{\ell }\left(t\right)-Y_{s}\left(t\right)∥)=\left\{\begin{array}{c} E,\;\mathrm{if}\;∥X_{\ell }\left(t\right)-Y_{s}\left(t\right)∥\leq R_{E},\\ k,\;\mathrm{if}R_{k+1}<∥X_{\ell }\left(t\right)-Y_{s}\left(t\right)∥\leq R_{k},k=1,\cdots ,E-1,\\ 0,\;\mathrm{otherwise}, \end{array}\right. \end{array}$$

where $R_{E}<R_{E-1}<\cdots <R_{1}$. Define a *charging range* as the maximum distance that a node receives at least one unit of energy from the connected WCS. Given the above recharging mechanism, the charging range is equivalent to $R_{1}$. The time required to transfer energy from a WCS to a node is extremely short compared to one slot. This means that the contact duration is long enough to deliver up to *E* units of energy under finite speed. It is a reasonable assumption because the maximum power transfer rate of magnetic resonance coupling is 12 (in Watts) [36], whereas that of an IoT device is 23 (in dBm), approximately 0.2 (in Watts).

The battery of each node is recharged by one of WCSs. When a node is in the coverage of multiple WCSs, it is assumed to receive energy from one of them due to the practical alignment technique limitation. The maximum battery capacity of each node is set to *L* units of energy. If the sum of residual and recharged energy are larger than *L*, a node stores up to *L* units of energy, and the remaining is thrown out. A WCS can recharge up to *u* nodes within one slot using the technique of tracking resonance frequencies. For example, it is experimentally shown in [37,38] that up to two devices can be charged by using the technique of the said resonant frequency splitting and load balancing, respectively. When there are more than *u* nodes within the coverage, the WCS randomly selects *u* nodes among them.

Each WCS always monitors its own remaining energy. If the remaining energy is below a certain level, it communicates with an operator station by using its communication module. The operator station then sends the charging vehicle, which recharges the WCS before its battery runs out. This means that all WCSs always have sufficient energy.

## 3. Stochastic Modeling of Energy-Efficient Opportunistic Internet-of-Things

In this section, we design a two-dimensional Markov chain in which the horizontal and vertical state dimensions represent the residual energy and the distance to the nearest WCS, respectively. We first outline our Markov chain design, and then derive the steady state probabilities to determine the active probability $P_{\mathrm{on}}$ (3).

### 3.1. Two-Dimensional Markov Chain

The state space of the proposed two-dimensional Markov chain Ψ is given as follows:

(5) $$\begin{array}{c} \Psi =\left\{\left(e,d\right):0\leq e\leq L,\;0\leq d\leq M\right\}, \end{array}$$

where parameter *e* is the number of remaining units of energy, and *d* is a discrete number indicating the distance to the nearest WCS by the following rule:

(6) $$\begin{array}{c} d=\left\{\begin{array}{cc} 0, & \mathrm{if}\;\min_{s}∥X_{\ell }\left(t\right)-Y_{s}\left(t\right)∥\leq R_{1},\\ 1, & \mathrm{else}\;\mathrm{if}\;\min_{s}∥X_{\ell }\left(t\right)-Y_{s}\left(t\right)∥\leq R_{1}+v,\\ \;\;\vdots & \\ k, & \mathrm{else}\;\mathrm{if}\;\min_{s}∥X_{\ell }\left(t\right)-Y_{s}\left(t\right)∥\leq R_{1}+kv,\\ \;\;\vdots & \\ M, & \mathrm{otherwise}, \end{array}\right. \end{array}$$

where $\min_{s}∥X_{\ell }\left(t\right)-Y_{s}\left(t\right)∥$ represents the distance from node *ℓ* to the nearest WCS (in meters), and the charging coverage $R_{1}$ and node speed ν are specified in (1) and (4), respectively. The number *M* in (6) can be interpreted as the resolution of the Markov chain in the sense that larger *M* is able to express the trajectory of node *ℓ* more accurately. The number *d* is defined as a *relative distance* meaning that a physical distance (in meters) is normalized by node speed *v*.

**Figure 2:**
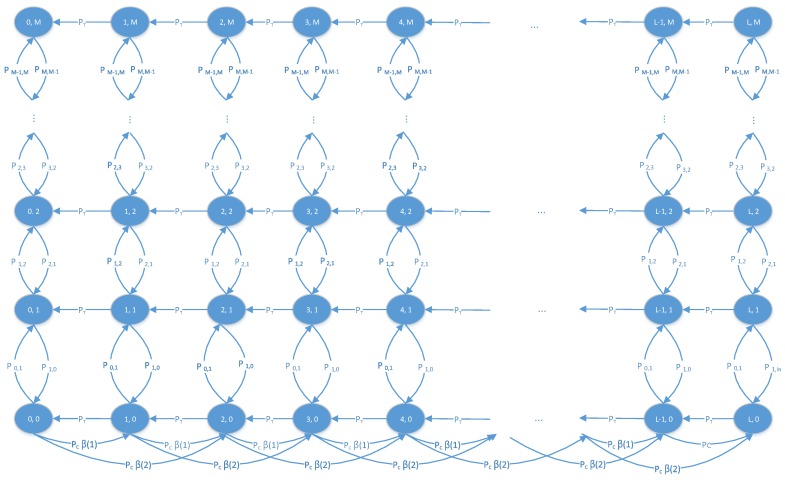
Two-dimensional Markov chain of which the horizontal and vertical state dimensions represent the number of remaining units of energy and the relative distance to the nearest WCS normalized by node speed, respectively.

Figure 2 represents an example of the two-dimensional Markov chain when WCS can deliver up to two units of energy to a node within one slot (E=2). The state transitions are explained as follows:

- **State transition by node mobility**: The state transitions to the up or down arise when the relative distance *d* (6) becomes shorter or longer, respectively. Let $P_{i,j}$ denote the probability that the relative distance *d* is changed from *a* to *b*, i.e.,
  (7) $$\begin{array}{c} P_{i,j}\left(t\right)=\mathrm{Pr}\left[d=j\;\mathrm{at}\;\mathrm{slot}\;t+1|d=i\;\mathrm{at}\;\mathrm{slot}\;t\right]. \end{array}$$
  The mobility model follows a time-invariant Markov process of which the transition probabilities are constant regardless of slot *t*, and $P_{i,j}\left(t\right)$ can be simply expressed as $P_{i,j}$ by omitting the index *t*. The exact form of $P_{i,j}$ is in Appendix A.1 with its derivation. All transition probabilities $P_{i,j}$ are constant regardless of the residual energy status.
- **State transition by data transmission**: The state transition to the left happens when node *ℓ* transmits a packet to one of neighbors nodes. Let $p_{t}$ denote a probability that an active node can transmit its packet as
  (8) $$\begin{array}{ccc} p_{t} & = & q\cdot \left[1-{\left\{1-\left(1-q\right)\frac{\pi r^{2}}{S}\right\}}^{n-1}\right]. \end{array}$$
  The detailed derivation is in [33]. Unless its residual energy *e* is zero, the transmission probability $p_{c}$ is constant regardless of the relative distance *d* (6).
- **State transition by energy charging**: The state transition to the right arises when the node is recharged by a WCS. This event only happens when the node is selected by one of WCSs is in the charging coverage, and these are only stipulated on the lowest state transition (d=0). Recall that each WCS can charge up to *u* nodes in a given slot. We define a charging probability $p_{c}$ as the probability that node *ℓ* becomes one of *u* selected nodes, i.e.,
  (9) $$\begin{array}{c} p_{c}=\frac{1-\gamma {\left(u,n\right)}^{m}}{1-{\left(1-\frac{\pi {R_{1}}^{2}}{S}\right)}^{m}}, \end{array}$$
  where $\gamma \left(u,n\right)=1-\frac{\pi {R_{1}}^{2}}{S}F\left(u-2;n-1,\frac{\pi {R_{1}}^{2}}{S}\right)-\frac{u}{n}\left(1-F(u-1;n,\frac{\pi {R_{1}}^{2}}{S}\right)$ and F(k;n,p)=∑i=0knipi(1−p)n−i is the *cumulative distribution function* (CDF) of the binomial distribution with parameters *k*, *n* and *p*. The derivation is given in Appendix A.2. The number of recharged units of energy depends on the distance to its associated WCS. Let β(k) denote a probability a node receives *k* units of energy as follows:
  $$\begin{array}{c} \beta \left(k\right)=\left\{\begin{array}{cc} \frac{{R_{k}}^{2}-{R_{k+1}}^{2}}{{R_{1}}^{2}}, & \;\mathrm{if}k=1,\cdots ,E-1,\\ \frac{{R_{k}}^{2}}{{R_{1}}^{2}}, & \;\mathrm{if}k=E. \end{array}\right. \end{array}$$
  A node in the charging coverage thus receives *k* units of energy with probability $p_{c}\beta \left(k\right)$.

### 3.2. Steady State Probability and Throughput

Let $\pi_{e,d}$ denote the steady state probability when the residual energy of node *ℓ* is *e* units of energy and the relative distance is *d*. Then, we make the following steady state vector $\pi =\left[\begin{array}{ccc} \pi_{0,0},\cdots \pi_{0,M}, & \pi_{1,0},\cdots \pi_{1,M},\cdots & \pi_{L,0},\cdots \pi_{L,M} \end{array}\right]$, which is partitioned according to the number of remaining units of energy, i.e., $\pi =\left[\begin{array}{cccc} \pi_{0} & \pi_{1} & \cdots & \pi_{L} \end{array}\right]$, where

(10) $$\begin{array}{cc} & \pi_{e}=\left[\begin{array}{cccc} \pi_{e,0} & \pi_{e,1} & \cdots & \pi_{e,M} \end{array}\right]. \end{array}$$

In order to derive π, we make the following balance equation:

(11) $$\begin{array}{c} \pi Q=0,\;\pi 1=1, \end{array}$$

where 1 is the column vector where every entity is one, and Q is the generating matrix of the corresponding Markov chain:

(12) $$\begin{array}{c} Q=\left(\begin{array}{ccccccccc} B_{0} & A_{2} & A_{3} & 0 & 0 & \cdots & 0 & 0 & 0\\ A_{0} & A_{1} & A_{2} & A_{3} & 0 & \cdots & 0 & 0 & 0\\ 0 & A_{0} & A_{1} & A_{2} & A_{3} & \cdots & 0 & 0 & 0\\ \vdots & \vdots & \vdots & \vdots & \vdots & \ddots & \vdots & \vdots \\ 0 & 0 & 0 & 0 & 0 & \cdots & A_{1} & A_{2} & A_{3}\\ 0 & 0 & 0 & 0 & 0 & \cdots & A_{0} & A_{1} & A_{2}+A_{3}\\ 0 & 0 & 0 & 0 & 0 & \cdots & 0 & A_{0} & A_{1}+A_{2}+A_{3} \end{array}\right). \end{array}$$

Its sub-matrices $B_{0}$, $A_{0}$, $A_{1}$, $A_{2}$ and $A_{3}$ are expressed as follows:

$$\begin{array}{c} B_{0}=\left(\begin{array}{ccccc} -P_{0,1}-p_{c} & P_{0,1} & 0 & \cdots & 0\\ P_{1,0} & -P_{1,0}-P_{1,2} & P_{1,2} & \cdots & 0\\ 0 & P_{2,1} & -P_{2,1}-P_{2,3} & \cdots & 0\\ \vdots & \vdots & \vdots & \ddots & \vdots \\ 0 & 0 & 0 & \cdots & -P_{M,M-1} \end{array}\right), \end{array}$$

$$A_{0}=\left(\begin{array}{ccc} p_{t} & \cdots & 0\\ \vdots & \ddots & \vdots \\ 0 & \cdots & p_{t} \end{array}\right)=p_{t}I,\;A_{1}=B_{0}-A_{0},A_{2}=\left(\begin{array}{ccc} p_{c}\beta (1) & \cdots & 0\\ \vdots & \ddots & \vdots \\ 0 & \cdots & 0 \end{array}\right),\;A_{3}=\left(\begin{array}{ccc} p_{c}\beta (2) & \cdots & 0\\ \vdots & \ddots & \vdots \\ 0 & \cdots & 0 \end{array}\right).$$

After solving the balance equation of (11), we can caculate the active probability $P_{\mathrm{on}}$ as

(13) $$\begin{array}{c} P_{\mathrm{on}}=\sum_{e=1}^{L}\pi_{e}1=1-\pi_{0}1. \end{array}$$

With (3), the throughput Λ is given as

(14) $$\begin{array}{c} \Lambda =\frac{1}{2}q\left(1-\pi_{0}1\right)\exp\left(-\frac{\pi }{4}q\left(1-\pi_{0}1\right)\right)\left(1-\exp\left(\frac{\pi }{4}\left(-1+q\right)\right)\right). \end{array}$$

## 4. Performance Evaluation of Energy-Efficient Opportunistic Internet-of-Things

Based on the aforementioned mathematical framework, this section attempts to analyze the effects of node speed on throughput in terms of inter-meeting time, battery capacity, and node density, each of which is verified by comparing Monte-Carlo simulations.

### 4.1. Inter-Meeting Time and Throughput

This subsection aims at analyzing the effect of node speed *v* on throughput Λ using the inter-meeting time defined as follows:

**Definition** **2.** *(Inter-meeting time) Consider that there are node ℓ and WCS s in the network. The inter-meeting time*$T_{I}$ is the interval between adjacent meeting events between node ℓ and WCS s:

(15) $$\begin{array}{c} T_{I}=\inf\left\{t\geq 0:Z_{t+k}=1|Z_{k}=1\right\}, \end{array}$$

*where $Z_{t}$ is an indicator to check whether a meeting event occurs between node ℓ and WCS s at time t. If $∥X_{\ell }\left(t\right)-Y_{s}\left(t\right)∥\leq R_{1}$, we set $Z_{t}$ to one. Otherwise, $Z_{t}=0$.*

The inter-meeting time $T_{I}$ is related to the energy starving period of the node because the node has no opportunity to receive energy until it meets one of WCSs. The stochastic features of $T_{I}$ is thus related to an energy provision process of an arbitrary node. Let P denote an *M* by *M* matrix of which the elements represents the transition probability $P_{i,j}$ (7) (1≤a,b≤M):

(16) $$\begin{array}{c} P=\left(\begin{array}{c} p_{1}\\ p_{2}\\ \vdots \\ p_{M} \end{array}\right)=\left(\begin{array}{ccccc} P_{1,1} & P_{1,2} & \cdots & P_{1,M-1} & P_{1,M}\\ P_{2,1} & P_{2,2} & \cdots & P_{1,M-1} & P_{2,M}\\ \vdots & \vdots & \ddots & \vdots & \vdots \\ P_{M,1} & P_{M,2} & \cdots & P_{M-1,M} & P_{M,M} \end{array}\right), \end{array}$$

where $p_{d}=\left(\begin{array}{ccccc} P_{d,1} & P_{d,2} & \cdots & P_{d,M-1} & P_{d,M} \end{array}\right)$. From P (16), we derive the stochastic distribution of inter-meeting time $T_{I}$ in the following Proposition:

**Proposition** **1.** *The complementary cumulative distribution function (CCDF) of the inter-meeting time $T_{I}$ is*

(17) $$\begin{array}{c} \mathrm{Pr}\left[T_{I}>t\right]=\sum_{i=1}^{M}\gamma_{i}\lambda_{i}^{t-1}, \end{array}$$

*where $\lambda_{i}$ is the $i^{th}$ eigenvalue of P (16) ($1>\lambda_{1}>\cdots >\lambda_{M}>0$). The coefficient $\gamma_{i}$ is*

$$\begin{array}{ccc} \gamma_{i} & = & p_{0}a_{i}b_{i}^{T}, \end{array}$$

where vectors $a_{i}$ and $b_{i}$ are the right-hand and left-hand eigenvectors of $\lambda_{i}$ such that $Pa_{i}=\lambda_{i}a_{i}$ and $b_{i}^{*}P=\lambda_{i}b_{i}^{*}$, respectively.

**Proof.**  See Appendix A.3. ☐

**Figure 3:**
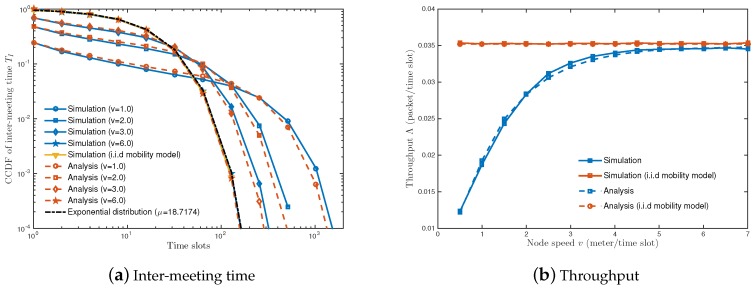
(**a**) The CCDF of inter-meeting time under different node speed *v* (meter/slot); (**b**) expected throughput as a function of node speed *v* (meter/slot). Parameters: Network size *S* = 400 (in meter^2^), battery capacity *L* = 10 (units of energy), the maximum number of simultaneous transferable nodes *u* = 1, the maximum transferable energy per slot *E* = 3 (in units of energy), the number of nodes *n* = 10, and the number of WCSs *m* = 1.

Figure 3a shows the CCDFs of inter-meeting time $T_{I}$. We use the energy transfer efficiency function in [39], i.e., $\tau \left(x\right)=-0.0958x^{2}-0.0377x+1.0$, which is obtained through the curve fitting of the experimental results of [40]. We numerically measure the inter-meeting time $T_{I}$ by changing the node speed as v=1, 2, 3 and 6 (meters/slot). When the length of one slot is set to a second, the concerned sets of speed represent the cases of stationary, walking, slow vehicle and fast vehicle, respectively [41]. It is shown that higher node speed *v* reduces the number of lengthy inter-meeting times. A node with faster speed can reach the charging coverage of the WCS within a few slots, reducing the occurrence of lengthy inter-meeting times. A node with higher speed enables to move a new location far away from its previous one. In other words, the event of meeting WCS depends on the ratio of the charging coverage to the network area, i.e., $\frac{1}{\mu }=\frac{\pi {R_{1}}^{2}}{S^{2}}\approx 0.053$ as does the i.i.d. mobility model. With increased node speed, the distribution converges to that of the i.i.d. mobility model following the exponential distribution with parameter μ≈18.7174. It is verifed by simulation that the analytic result in Proposition 1 follows similar tendencies of practical mobility models e.g., Brownian motion and random waypoint (See Appendix A.4).

The CCDF of $T_{I}$ of (17) is the sum of powered eigenvalues with the exponent *t*. As *t* becomes larger, it is simplified by the largest eigenvalue $\lambda_{1}$ because other terms decay faster:

(18) $$\begin{array}{c} \mathrm{Pr}\left[T_{I}>t\right]\approx \lambda_{1}^{t}. \end{array}$$

The eigenvalue $\lambda_{1}$ is called the *spectral radius* of matrix P (16). As the spectral radius becomes smaller, the approximated CCDF (18) decreases much faster in the regime of large *t*. This indicates that lengthy inter-meeting times are infrequent when $\lambda_{1}$ is small. In Table 1, we summarize this spectral radius $\lambda_{1}$ as a function of node speed *v* and show that $\lambda_{1}$ is a non-increasing function of node speed *v*. Consequently, higher node speed decreases spectral radius $\lambda_{1}$ and produces fewer occurrences of lengthy inter-meeting times.

The above feature of the inter-meeting time affects the energy provision process as shown in Figure 3b. When node speed *v* is 0.5 (meters/slot), the throughput Λ is nearly one-third of that of the i.i.d. mobility model. A node is unable to receive energy for a long time due to frequent lengthy inter-meeting time and remains in an inactive state. This results in the decrease in throughput. As *v* increases, on the other hand, the inter-meeting time decreases. This leads to the reduction in energy-starving period and the improvement of throughput.

### 4.2. Battery Capacity and Throughput

Consider a slow-moving node who stays in the charging coverage for a long duration. The node can receive energy continuously from the connected WCS. Nevertheless, the node is unable to save more than *L* units of energy due to the battery capacity constraint. In other words, the node can remain active longer with larger battery capacity. To explain the phenomenon, we make the following proposition.

**Proposition** **2.** *When the battery capacity L becomes infinite, the throughput of an energy-constrained network *Λ* becomes independent of node speed v as*

(19) $$\begin{array}{c} \Lambda =\frac{q}{2}\rho \left(1-\exp\left(\frac{\pi (q-1)}{4}\right)\right)\cdot \exp\left(-\frac{\pi }{4}q\cdot \rho \right), \end{array}$$

*where*

(20) $$\begin{array}{c} \rho =\min\left[1,\frac{p_{c}}{p_{t}}\left\{1-{\left(1-\frac{\pi {R_{1}}^{2}}{S}\right)}^{m}\right\}\left(\sum_{i=k}^{E}k\beta \left(k\right)\right)\right]. \end{array}$$

**Proof.**  See Appendix A.5. ☐

**Figure 4:**
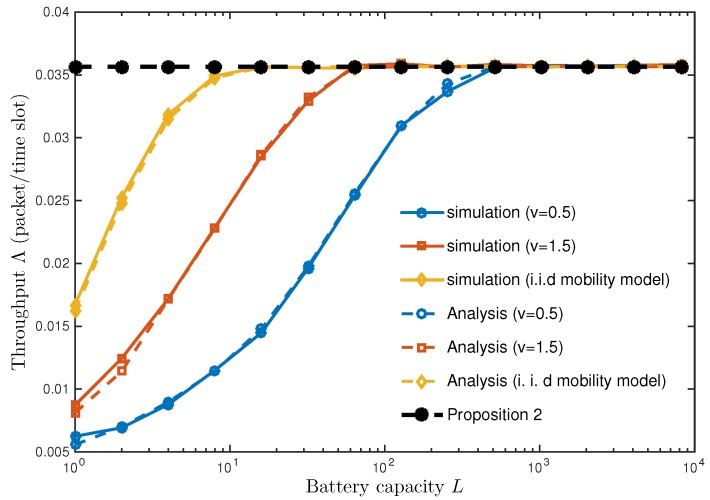
Throughput vs. battery capacity *L*. The same parameter setting as in Figure 3 is used unless specified.

Figure 4 represents the throughput Λ as a function of battery capacity *L*. As *L* increases, Λ increases and converges as mentioned in Proposition 2 (see the black dotted line). A noticeable point is that Proposition 2 is achievable under a finite battery capacity. If a node can store enough energy to sustain the inter-meeting time, it remains in an active state and achieves the throughput in Proposition 2. We calculate the mean of the inter-meeting time $E[T_{I}]$ from (18) and the spectral radius $\lambda_{1}$ in Table 1:

(21) $$\begin{array}{c} E\left[T_{I}\right]=\sum_{t=0}^{\infty }\mathrm{Pr}\left[T_{I}>t\right]\approx \sum_{t=0}^{\infty }{\left(\lambda_{1}\right)}^{t}=\frac{1}{1-\lambda_{1}}. \end{array}$$

When the battery capacity *L* is no less than $E[T_{I}]$, the throughput Λ becomes the same as that in Proposition 2 (19). For example, when node speed *v* is 0.5 or 1.5 (meters/slot), its spectral radius $\lambda_{1}$ is 0.9985 or 0.9903 (see Table 1) and its corresponding $E[T_{I}]$ becomes 666.67 or 103.09, respectively. As a result, a battery capacity larger than $E[T_{I}]$ is understood as a necessary condition to achieve the upper bound in Proposition 2.

### 4.3. Node Density and Throughput

Since the seminal work by Grossglauser and Tse [35], investigating the relationship between throughput Λ and node density *n* has been the most fundamental issue with mobile networks; therefore, the impact of irregular energy provision due to low node speed has not yet been studied. In this subsection, we investigate this effect through some numerical evaluations and the following throughput scaling law.

**Proposition** **3.** *The scaling law of the throughput* Λ *is:*

(22) $$\begin{array}{c} \Lambda =\Theta \left(\min\left(1,\frac{m}{n}\right)c^{\min\left(1,\frac{m}{n}\right)}\right), \end{array}$$

*where $0<e^{-\frac{\pi \cdot u}{4\cdot a}}<c\leq e^{-\frac{\pi \cdot u}{4\cdot a}\left(\sum_{k=1}^{E}k\beta \left(k\right)\right)}<1$, and $a=1-e^{-\frac{\pi }{4}\left(1-q\right)}$.*

**Proof.**  See Appendix A.6. ☐

Proposition 3 indicates that the throughput Λ is a function of the ratio of the number of WCSs *m* and the number of nodes *n*, and independent of node speed *v*. A node with low speed receives energy from WCSs irregularly, yielding the decrease of throughput. Compared with fast-moving one, it needs more WCSs to maintain the same throughput. As the network becomes denser, however, the penalty due to slow speed disappears and we only consider the ratio $\frac{m}{n}$ when installing WCSs. In order to achieve the constant throughput of Θ(1) as in [35], for example, Θ(n) WCSs is required regardless of node speed.

**Figure 5:**
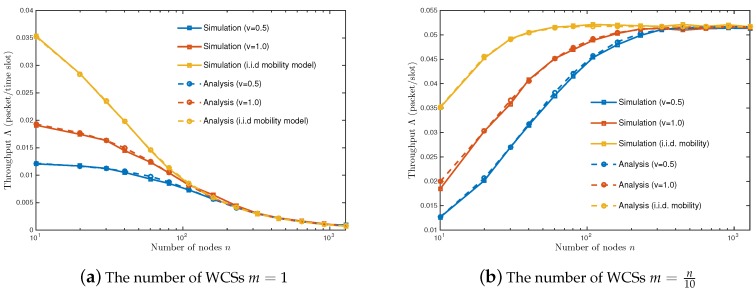
Throughput vs. node density *n*. The same parameter setting as in Figure 3 is used unless specified.

Note that the scaling law in Proposition 3 of (22) is the same as that of the i.i.d. mobility model in [33]. Figure 5 shows that the throughput Λ always converges to that of the i.i.d. mobility model as the number of nodes *n* increases. This implies that a high node density makes nodes look as if they are moving at a fast speed in the sense that the i.i.d. mobility model allows a node to increase moving speed *v* up to the network size. When calculating the throughput of a dense mobile network with WPT, it is a reasonable assumption that nodes move according to the i.i.d. mobility model.

## 5. Conclusions

In this paper, we have investigated the throughput of an energy-constrained opportunistic IoT network where WCSs are deployed to recharge IoT nodes when they are in the charging coverage. Given the network architecture, the energy provision pattern follows the speed of the corresponding node. Namely, a slow-moving node outside a WCS’s charging coverage waits a long time for energy supply from WCSs, whereas a fast-moving one can receive energy from a WCS within a short interval. The analytical and numerical results have shown that this distinct energy provisioning difference leads to the throughput gap between slow- and fast-moving nodes especially when the battery capacity is finite and IoT nodes are sparsely deployed. This finding provides useful guidelines for designing energy-efficient opportunistic IoT networks. First, a WCS should prioritize the charging opportunity of nodes depending on its speed to improve energy provision efficiency. Second, the battery capacity of an IoT node can be minimized by predicting its speed. Finally, the deployment strategy of WCSs should be different depending on node speed such that a relatively less number of WCSs per node is enough to support the whole nodes in area of high mobility, e.g., motorway, while more WCSs per node are required to guarantee the same throughput in area of slow mobility, e.g., pedestrian way.

The current work can be extended in several directions. In this work, we consider the simple mobility model where each node moves without preference. In practical, on the other hand, people are likely to visit some popular places frequently, making it very challenging to supply enough energy due to the relatively high node density in the area. Next, considering vehicular scenarios such that safety-information is disseminated based on *vehicle-to-everything* (V2X) communication is aligned with the recent trend of wireless communications. Moreover, considering the economic aspect of WCSs is another interesting avenue for future research.

## Figures and Tables

**Table 1:** Spectral radius $\lambda_{1}$ as a function of node speed *v* (meters/slot). The same parameter setting is used as Figure 3.

	*v* = 0.5	*v* = 1.0	*v* = 1.5	*v* = 2.0	*v* = 2.5	*v* = 3.0	*v* = 3.5	*v* = 4.0	*v* = 4.5	*v* = 5.0	*v* = 5.5	*v* = 6.0
$\lambda_{1}$	0.9985	0.9953	0.9903	0.9845	0.9780	0.9714	0.9649	0.9585	0.9534	0.9492	0.9471	0.9457

**Figure A1:**
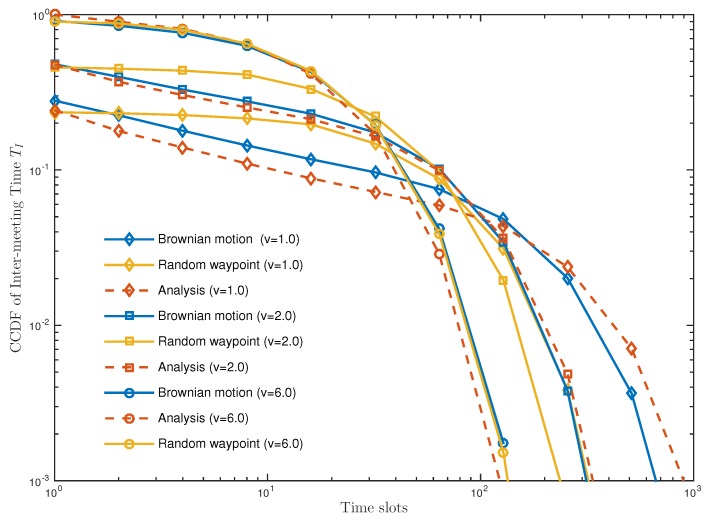
The CCDF of inter-meeting time of practical mobility models. The same parameter setting as in Figure 3 is used unless specified.

## Abbreviations

WPT	Wireless power transfer
IoT	Internet-of-things
WCS	Wireless charging station
D2D	Device-to-device
RF	Radio-frequency
WCV	Wireless charging vehicle
i.i.d.	Independent and identically distributed
QoEP	Quality of energy provisioning
MCS	Modulation and coding scheme
CDF	Cumulative distribution function
CCDF	Complementary cumulative distribution function
V2X	Vehicular-to-everything
BM	Brownian motion
RWP	Random way point
BMAP	Batch Markovian arrival process
QBD	Quasi-birth-death

## Appendix A

### Appendix A.1. Derivation of Transition Probability P_i,j_ *(7)*

Let $D_{t}$ denote the distance between a node and a WCS at time *t*. Since nodes and WCSs are uniformly distributed in a torus area, the conditional probability that $D_{t+1}$ is smaller than or equal to $x_{2}$ given $D_{t}=x_{1}$ is

(A1) $$\begin{array}{c} \mathrm{Pr}\left[D_{t+1}\leq x_{2}|D_{t}=x_{1}\right]=\left\{\begin{array}{cc} 0, & \mathrm{if}v+x_{2}<x_{1}\mathrm{or}v-x_{2}>x_{1},\\ \frac{\arccos\left(\frac{v^{2}+{x_{1}}^{2}-x_{2}^{2}}{2vx_{1}}\right)}{\pi }, & \mathrm{if}|v-x_{2}|\leq x_{1}<v+x_{2},\\ 1, & \mathrm{if}x_{2}-v>x_{1}, \end{array}\right. \end{array}$$

which is based on the fact that nodes and WCSs are uniformly distributed in a torus area. From the conditional probability (A1), we derive the joint cumulative distribution function (CDF) that $D_{t}$ is smaller than or equal to $x_{1}$, and $D_{t+1}$ is smaller than or equal to $x_{1}$:

(A2) $$\begin{array}{c} \mathrm{Pr}\left[D_{t}\leq x_{1},D_{t+1}\leq x_{2}\right]=\int_{0}^{x_{1}}\mathrm{Pr}\left[D_{t+1}\leq x_{2}|D_{t}=x\right]f_{D_{t}}\left(x\right)dx. \end{array}$$

Using (A2), we calculate the following joint probability:

(A3) $$\begin{array}{cc} cc & \mathrm{Pr}\left[x_{1}\leq D_{t}\leq x_{2},x_{3}\leq D_{t+1}\leq x_{4}\right]=\mathrm{Pr}\left[D_{t}\leq x_{2},D_{t+1}\leq x_{4}\right]\\ & -\left[D_{t}\leq x_{1},D_{t+1}\leq x_{4}\right]-\mathrm{Pr}\left[D_{t}\leq x_{2},D_{t+1}\leq x_{3}\right]+\mathrm{Pr}\left[D_{t}\leq x_{1},D_{t+1}\leq x_{3}\right]. \end{array}$$

By inserting the boundary values of the *i* and *j* states in (6) into $x_{1}$, $x_{2}$, $x_{3}$ and $x_{4}$ in (A3), we can derive the joint probability $\alpha_{i,j}$ that relative distances *d* of (6) in slot *t* and t+1 are *i* and *j*, respectively. For example, to calculate $\alpha_{0,1}$, we set $x_{1}=0$, $x_{2}=R_{1}$, $x_{3}=R_{1}$ and $x_{4}=R_{1}+v$.

Define $A_{a,b}$ as the joint CCDF that distances $d_{t}$ and $d_{t+1}$ are respectively no less than *a* and *b* when the number of WCSs *m* is one, which is expressed as the sum of $\alpha_{i,j}$, i.e., $A_{a,b}=\sum_{i=a}^{M}\sum_{j=b}^{M}\alpha_{i,j}.$ Noting that each location of WCSs is independent, we derive $P_{i,j}$ in terms of $A_{a,b}$ as follows:

- If i=0,
  $$\begin{array}{c} P_{0,j}=\left\{\begin{array}{cc} 1-\frac{{A_{0,1}}^{m}-{A_{1,1}}^{m}}{1-{\left(1-\frac{\pi {R_{1}}^{2}}{S}\right)}^{m}}, & \;\;\;\;\;\;\;\mathrm{if}j=0,\\ \frac{{A_{0,1}}^{m}-{A_{1,1}}^{m}}{1-{\left(1-\frac{\pi {R_{1}}^{2}}{S}\right)}^{m}}, & \;\;\;\;\;\;\;\mathrm{if}j=1,\\ 0, & \;\;\;\;\;\;\;\mathrm{otherwise}. \end{array}\right. \end{array}$$
- If 0<i<M,
  $$\begin{array}{c} P_{i,j}=\left\{\begin{array}{cc} 1-\frac{{A_{i,i}}^{m}-{A_{i+1,i}}^{m}}{{\left\{1-\frac{\pi {\left(R_{1}+\left(i-1\right)v\right)}^{2}}{S}\right\}}^{m}-{\left\{1-\frac{\pi {\left(R_{1}+iv\right)}^{2}}{S}\right\}}^{m}}, & \mathrm{if}j=i-1,\\ \frac{{A_{i,i}}^{m}-{A_{i+1,i}}^{m}-{A_{i+1,i}}^{m}+{A_{i+1,i+1}}^{m}}{{\left\{1-\frac{\pi {\left(R_{1}+\left(i-1\right)v\right)}^{2}}{S}\right\}}^{m}-{\left\{1-\frac{\pi {\left(R_{1}+iv\right)}^{2}}{S}\right\}}^{m}}, & \mathrm{if}j=i,\\ \frac{{A_{i,i+1}}^{m}-{A_{i+1,i+1}}^{m}}{{\left\{1-\frac{\pi {\left(R_{1}+\left(i-1\right)v\right)}^{2}}{S}\right\}}^{m}-{\left\{1-\frac{\pi {\left(R_{1}+iv\right)}^{2}}{S}\right\}}^{m}} & \mathrm{if}j=i+1,\\ 0, & \mathrm{otherwise}. \end{array}\right. \end{array}$$
- If i=M,
  $$\begin{array}{c} P_{M,j}=\left\{\begin{array}{cc} \frac{{A_{M,M-1}}^{m}-{A_{M,M}}^{m}}{{\left(1-\frac{\pi {\left(R_{1}+Mv\right)}^{2}}{S}\right)}^{m}}, & \;\;\;\;\;\;\mathrm{if}j=M-1,\\ \frac{{A_{M,M}}^{m}}{{\left(1-\frac{\pi {\left(R_{1}+Mv\right)}^{2}}{S}\right)}^{m}}, & \;\;\;\;\;\;\mathrm{if}j=M,\\ 0, & \;\;\;\;\;\;\mathrm{otherwise}. \end{array}\right. \end{array}$$

### Appendix A.2. Derivation of Charging Probability p_c_ *(9)*

Given that there are *h* WCSs within $R_{1}$ from a node, the probability that the node is charged by one of the WCSs $p_{c}\left(h\right)$ is

(A4) $$\begin{array}{cc} p_{c}\left(h\right) & =1-{\left[1-\sum_{\ell =0}^{n-1}\min\left[1,\frac{u}{\ell +1}\right]f\left(\ell ;n-1,\frac{\pi {R_{1}}^{2}}{S}\right)\right]}^{h}=1-\Gamma^{h}, \end{array}$$

where f(n;k,p) is the probability density function of the binomial distribution with parameters *n*, *k* and *p*, and

(A5) $$\begin{array}{c} \Gamma =1-F\left(u-2;n-1,\frac{\pi {R_{1}}^{2}}{S}\right)-\frac{u\left(1-F\left(u-1;n,\frac{\pi {R_{1}}^{2}}{S}\right)\right)}{n\pi R^{2}}. \end{array}$$

The probability that there are *h* WCSs within $R_{1}$ from a node is $f\left(h;m,\frac{\pi {R_{1}}^{2}}{S}\right)$. Therefore, the charging probability $p_{c}$ is

(A6) $$\begin{array}{c} p_{c}=\frac{\sum_{h=1}^{m}p_{c}\left(h\right)f\left(h;m,\frac{\pi {R_{1}}^{2}}{S}\right)}{1-{\left(1-\frac{\pi {R_{1}}^{2}}{S}\right)}^{m}}. \end{array}$$

The denominator of (A6) represents the probability that the node is in one of the WCS’s charging coverage. After substituting (A4) into (A6), the charging probability $p_{c}$ becomes

(A7) ccpc=1−∑h=1mmhAπR12Sh1−πR12Sm−h1−1−πR12Sm=1−∑h=0mmhAπR12Sh1−πR12Sm−h1−1−πR12Sm=1−AπR12S+1−πR12Sm1−1−πR12Sm.

Plugging (A5) into (A7) completes the proof.

### Appendix A.3. Proof of Proposition 1

According to [42], the CCDF of $T_{I}$ is $\mathrm{Pr}\left[T_{I}>t\right]=p_{0}P^{t-1}1$. Assume that matrix P (16) is invertible (It is a reasonable assumption that the transition probability, expressed as a row vector in P, is independent of the current location status *d* (6) unless speed is infinite, and P is likely to be a full rank matrix guaranteeing the existence of *M* eigenvalues. It is also verified numerically under numerous combinations of parameter settings.), and it can be diagonalized as follows:

(A8) $$\begin{array}{c} P=VDV^{-1}=\left(\begin{array}{cccc} a_{1} & a_{2} & \cdots & a_{M} \end{array}\right)\left(\begin{array}{cccc} \lambda_{1} & 0 & \cdots & 0\\ 0 & \lambda_{2} & \cdots & 0\\ \vdots & \vdots & \ddots & \vdots \\ 0 & 0 & \cdots & \lambda_{M} \end{array}\right)\left(\begin{array}{c} b_{1}^{T}\\ b_{2}^{T}\\ \vdots \\ b_{M}^{T} \end{array}\right). \end{array}$$

Therefore, $P^{t-1}$ is

(A9) $$\begin{array}{c} P^{t-1}=V{\left(D\right)}^{t-1}V^{-1}=\sum_{i=1}^{M}G_{i}\cdot {\left(\lambda_{i}\right)}^{t-1}, \end{array}$$

where $G_{i}=a_{i}b_{i}^{T}$ are M×M matrices of which the sum is an identity matrix $\left(\sum_{i=1}^{M}G_{i}=I\right)$. From (A9), $\mathrm{Pr}\left[T_{I}>t\right]$ is given as

$$\begin{array}{ccc} \mathrm{Pr}\left[T_{I}>t\right] & = & \sum_{i=1}^{M}p_{0}G_{i}1{\lambda_{i}}^{t-1}=\sum_{i=1}^{M}\gamma_{i}\cdot {\lambda_{i}}^{t-1}. \end{array}$$

Matrix P (16) is called a sub-stochastic matrix because every row sum is 1 except the first one due to a strictly positive transition probability $P_{1,0}$. Noting that every eigenvalue of an irreducible sub-stochastic matrix is less than 1, $\lambda_{i}$ should be smaller than one for every *i*.

### Appendix A.4. Comparison with Practical Mobility Models

We measure the inter-meeting time of *Brownian motion* (BM) and *random way point* (RWP), which is shown in Figure A1 that the analytic result tends to overestimate the frequency of long inter-meeting than the practical models due to its constant moving speed. Nevertheless, the overall tendencies are quite similar especially when speed becomes high.

### Appendix A.5. Proof of Proposition 2

As L→∞, this Markov process (12) becomes *batch Markovian arrival process* (BMAP), of which the stochastic process can be described by the mean steady-state arrival rate $\bar{\lambda }$ derived as follows. First, an infinite generator D is given as

(A10) $$\begin{array}{c} D=B_{0}+A_{2}+A_{3}+\cdots +A_{E-1}=\left(\begin{array}{ccccc} -P_{0,1} & P_{0,1} & 0 & \cdots & 0\\ P_{1,0} & -P_{1,0}-P_{1,2} & P_{1,2} & \cdots & 0\\ 0 & P_{2,1} & -P_{2,1}-P_{2,3} & \cdots & 0\\ \vdots & \vdots & \vdots & \ddots & \vdots \\ 0 & 0 & 0 & \cdots & -P_{M,M-1} \end{array}\right). \end{array}$$

Let $\phi_{k}$ denote steady state probability that the relative distance *d* is *k*. We make the following row vector ϕ as

$$\begin{array}{c} \phi =\left[\begin{array}{ccccc} \phi_{0}, & \phi_{1}, & \phi_{2}, & \cdots , & \phi_{M} \end{array}\right]=\left[\begin{array}{ccccc} \sum_{j=0}^{L}\pi_{0,j}, & \sum_{j=0}^{L}\pi_{1,j}, & \sum_{j=0}^{L}\pi_{2,j}, & \cdots , & \sum_{j=0}^{L}\pi_{M,j} \end{array}\right], \end{array}$$

which can be derived by solving the following equations.

(A11) $$\begin{array}{c} \phi D=0,\;\phi 1=1. \end{array}$$

From (A11), we have

(A12) $$\begin{array}{c} \phi_{k}=\left\{\begin{array}{cc} 1-{\left(1-\frac{\pi {R_{1}}^{2}}{S}\right)}^{m}, & \mathrm{if}k=0,\\ {\left(1-\frac{\pi {\left(R_{1}+Mv\right)}^{2}}{S}\right)}^{m}, & \mathrm{if}k=M,\\ {\left\{1-\frac{\pi {\left(R_{1}+\left(k-1\right)v\right)}^{2}}{S}\right\}}^{m}-{\left\{1-\frac{\pi {\left(R_{1}+kv\right)}^{2}}{S}\right\}}^{m}, & \mathrm{otherwise}. \end{array}\right. \end{array}$$

Finally, we derive the mean steady-state arrival rate $\bar{\lambda }$ from (A12),

$$\begin{array}{c} \bar{\lambda }=\phi \left(\sum_{k=1}^{E}kA_{k+1}\right)1=p_{c}\left\{1-{\left(1-\frac{\pi {R_{1}}^{2}}{S}\right)}^{m}\right\}\left(\sum_{k=1}^{E}k\beta \left(k\right)\right). \end{array}$$

If $\bar{\lambda }<p_{t}$, the active probability $P_{\mathrm{on}}$ is

(A13) $$\begin{array}{c} P_{\mathrm{on}}=\frac{\bar{\lambda }}{p_{t}}=\frac{p_{c}}{p_{t}}\left\{1-{\left(1-\frac{\pi {R_{1}}^{2}}{S}\right)}^{m}\right\}\left(\sum_{k=1}^{E}k\beta \left(k\right)\right). \end{array}$$

Otherwise, $P_{\mathrm{on}}=1$. After inserting (A13) into (3), we complete the proof.

### Appendix A.6. Proof of Proposition *3*

For the first step, we check the ratio $\frac{p_{c}}{p_{t}}$ as the number *n* increases,

(A14) $$\begin{array}{cc} \frac{p_{c}}{p_{t}} & \approx \frac{1-{\left(1-\frac{u}{n}\right)}^{m}}{q\left(1-{\left(1-\frac{\pi R_{1}^{2}}{S}\right)}^{m}\right)\left(1-e^{-\frac{\pi }{4}\left(1-q\right)}\right)}=\left\{\begin{array}{cc} \frac{m}{n}\frac{u}{q\left(1-{\left(1-\frac{\pi R_{1}^{2}}{S}\right)}^{m}\right)\left(1-e^{-\frac{\pi }{4}\left(1-q\right)}\right)} & \mathrm{if}m=O\left(n\right),\\ \frac{1}{q\left(1-{\left(1-\frac{\pi R_{1}^{2}}{S}\right)}^{m}\right)\left(1-e^{-\frac{\pi }{4}\left(1-q\right)}\right)}, & \mathrm{otherwise}. \end{array}\right. \end{array}$$

We already proved the upper bound in Proposition 2 (19) as follows:

(A15) $$\begin{array}{ccc} \Lambda \leq \Lambda_{\mathrm{upper}} & = & \Theta \left(\min\left(1,\frac{m}{n}\right){c_{1}}^{\min\left(1,\frac{m}{n}\right)}\right), \end{array}$$

where $c_{1}=e^{-\frac{\pi \cdot u}{4\cdot a}\left(\sum_{k=1}^{E}k\beta \left(k\right)\right)}$.

In order to derive the lower bound, consider that each WCS only delivers one unit of energy to a node in a slot (E=1). Since the submatrices $A_{2}$ and $A_{3}$ in the generating matrix Q (12) is null matrices, the Markov process becomes finite *Quasi-Birth-Death* (QBD) Process. In [43], the authors showed that the steady state probability vector $\pi_{k}$ (10) of finite QBD can be expressed in a matrix geometric form:

(A16) $$\begin{array}{c} \pi_{k}=v_{1}{R_{1}}^{k}+v_{2}{R_{2}}^{L-k}. \end{array}$$

Here, matrices $R_{1}$ and $R_{2}$ are

(A17) $$\begin{array}{c} R_{1}=-A_{2}{\left(A_{1}+\eta A_{0}\right)}^{-1}, \end{array}$$

(A18) $$\begin{array}{c} R_{2}=-A_{0}{\left(A_{1}+A_{0}G\right)}^{-1}, \end{array}$$

where η is the spectral radius of $R_{1}$, and G is the square matrix that the every element of the first column is one and the others are zero. Detailed derivations of $R_{1}$ and $R_{2}$ are in [44]. Row vectors $v_{1}$ and $v_{2}$ satisfy the following conditions:

(A19) $$\begin{array}{c} \left[\begin{array}{cc} v_{1} & v_{2} \end{array}\right]\left[\begin{array}{cc} B_{1}+R_{1}A_{0} & {R_{1}}^{L-1}\left(A_{0}+R_{1}\left(A_{0}+B_{0}\right)\right)\\ {R_{2}}^{L-1}\left(R_{2}B_{0}+A_{0}\right) & A_{0}+A_{1}+R_{2}A_{0} \end{array}\right]=0, \end{array}$$

(A20) $$\begin{array}{c} \left(v_{1}\sum_{i=0}^{L}{R_{1}}^{i}+v_{1}\sum_{i=0}^{L}{R_{1}}^{i}\right)1=1. \end{array}$$

The boundary condition (A19) is derived by inserting (A16) into the first and last columns of the balance Equation (11), and condition (A20) means the summation of the entire steady state probabilities is one:

(A21) $$\begin{array}{c} \begin{array}{lll} X_{1} & = & \left|\begin{array}{cc} \frac{p_{t}}{p_{c}}\frac{P_{0,1}+p_{c}}{P_{1,0}}+1 & \frac{p_{t}}{p_{c}}\frac{P_{0,1}+p_{c}}{P_{1,0}}\frac{P_{1,2}}{P_{2,1}}\\ \frac{p_{t}}{p_{c}}\frac{P_{0,1}+p_{c}}{P_{1,0}}+1 & \frac{p_{t}}{p_{c}}\frac{P_{0,1}+p_{c}}{P_{1,0}}\frac{P_{1,2}}{P_{2,1}}+\frac{p_{t}}{P_{2,1}}+1 \end{array}\right|,\;X_{2}=\left|\begin{array}{cc} \frac{p_{t}}{p_{c}}\frac{P_{0,1}}{P_{1,0}} & \frac{p_{t}}{p_{c}}\frac{P_{0,1}}{P_{1,0}}\frac{P_{1,2}}{P_{2,1}}\\ \frac{p_{t}}{p_{c}}\frac{P_{0,1}+p_{c}}{P_{1,0}}+1 & \frac{p_{t}}{p_{c}}\frac{P_{0,1}+p_{c}}{P_{1,0}}\frac{P_{1,2}}{P_{2,1}}+\frac{p_{c}}{P_{2,1}}+1 \end{array}\right|,\; \end{array}\\ \begin{array}{lll} X_{3} & = & \left|\begin{array}{cc} \frac{p_{t}}{p_{c}}\frac{P_{0,1}}{P_{1,0}} & \frac{p_{t}}{p_{c}}\frac{P_{0,1}}{P_{1,0}}\frac{P_{1,2}}{P_{2,1}}\\ \frac{p_{t}}{p_{c}}\frac{P_{0,1}+p_{c}}{P_{1,0}}+1 & \frac{p_{t}}{p_{c}}\frac{P_{0,1}+p_{c}}{P_{1,0}}\frac{P_{1,2}}{P_{2,1}} \end{array}\right|, \end{array} \end{array}$$

where $\left|\begin{array}{cc} a & b\\ c & d \end{array}\right|=ad-bc$.

From the Equation (A16), the active probability $P_{\mathrm{on}}$ (13) is rewritten as follows:

(A22) $$\begin{array}{c} P_{\mathrm{on}}=1-\left(v_{1}+v_{2}{R_{2}}^{L}\right)1. \end{array}$$

Since the active probability $P_{\mathrm{on}}$ is a non-decreasing function of the battery capacity *L*, we make the following inequality condition:

(A23) $$\begin{array}{c} P_{\mathrm{on}}\geq 1-\left(v_{1}+v_{2}R_{2}\right)1=\left(v_{1}\sum_{i=0}^{1}{R_{1}}^{i}+v_{1}\sum_{i=0}^{1}{R_{1}}^{i}\right)1-\left(v_{1}+v_{2}R_{2}\right)1=\left(v_{1}R_{1}+v_{2}\right)1. \end{array}$$

All elements in matrix $R_{1}$ is zero except the first row, and the first element of $v_{1}$ is zero. Therefore, $v_{1}R_{1}$ becomes zero and the above inequality (A23) becomes

(A24) $$\begin{array}{ccc} P_{\mathrm{on}} & \geq & v_{2}1=\sum_{i=0}^{M}v_{2,i}\geq v_{2,0}. \end{array}$$

From the condition (A19), we make the following relations between $v_{1}$ and $v_{2}$,

(A25) $$\begin{array}{c} v_{1}=-v_{2}\left(R_{2}+p_{t}B_{0}^{-1}\right), \end{array}$$

(A26) $$\begin{array}{c} v_{1}+v_{2}\left(I+R_{2}\right)=\phi . \end{array}$$

After inserting (A25) into (A26), we derive $v_{2}$ as

(A27) $$\begin{array}{c} v_{2}=\phi {\left(I-p_{t}B_{0}^{-1}\right)}^{-1}. \end{array}$$

According to numerical verifications, it is checked that $v_{2,0}$ is an increasing function of the resolution factor *M*. When M=3, the vector $v_{2}$ becomes:

(A28) $$\begin{array}{c} v_{2,0}=\phi \frac{1}{\left(\frac{p_{t}}{p_{c}}+1\right)X_{1}-\frac{p_{t}}{p_{c}}X_{2}+\frac{p_{t}}{p_{c}}X_{3}}\left(\begin{array}{c} X_{1}\\ -X_{2}\\ X_{3} \end{array}\right)=\frac{\phi_{1}X_{1}-\phi_{2}X_{2}+\phi_{3}X_{3}}{\left(\frac{p_{t}}{p_{c}}+1\right)X_{1}-\frac{p_{t}}{p_{c}}X_{2}+\frac{p_{t}}{p_{c}}X_{3}}>\frac{p_{c}}{p_{t}}\frac{\left(\phi_{1}-\phi_{2}\frac{X_{2}}{X_{1}}+\phi_{3}\frac{X_{3}}{X_{1}}\right)}{2}, \end{array}$$

where $X_{1}$, $X_{2}$ and $X_{3}$ are described in (A21). As *n* increases, the ratios $\frac{X_{2}}{X_{1}}$ and $\frac{X_{2}}{X_{1}}$ reduce to zero. From the inequalities (A24) and (A28), the active probability $P_{\mathrm{on}}$ is

(A29) $$\begin{array}{ccc} P_{\mathrm{on}} & > & \frac{p_{c}}{p_{t}}\frac{\phi_{1}}{2}=\frac{p_{c}}{p_{t}}\frac{\left\{1-{\left(1-\frac{\pi {R_{1}}^{2}}{S}\right)}^{m}\right\}}{2}. \end{array}$$

We can derive the lower bound of the throughput Λ as

(A30) $$\begin{array}{c} \Lambda >\Lambda_{\mathrm{lower}}=\Theta \left(\min\left(1,\frac{m}{n}\right){c_{2}}^{\min\left(1,\frac{m}{n}\right)}\right), \end{array}$$

where $c_{2}=e^{-\frac{\pi \cdot u}{8\cdot a}}$. From the upper bound (A15) and lower bound (A30), we complete the proof.
